# Accessibility, affordability and use of health services in an urban area in South Africa

**DOI:** 10.4102/curationis.v38i1.102

**Published:** 2015-03-10

**Authors:** Ethelwynn L. Stellenberg

**Affiliations:** 1 Division of Nursing, Faculty of Medicine and Health Sciences, Stellenbosch University, South Africa

## Abstract

**Background:**

Inequalities in healthcare between population groups of South Africa existed during the apartheid era and continue to exist both between and within many population groups. Accessibility and affordability of healthcare is a human right.

**Objectives:**

The aim of the study was to explore and describe accessibility, affordability and the use of health services by the mixed race (coloured) population in the Western Cape, South Africa.

**Method:**

A cross-sectional descriptive, non-experimental study with a quantitative approach was applied. A purposive convenient sample of 353 participants (0.6%) was drawn from a population of 63 004 economically-active people who lived in the residential areas as defined for the purpose of the study. All social classes were represented. The hypothesis set was that there is a positive relationship between accessibility, affordability and the use of health services. A pilot study was conducted which also supported the reliability and validity of the study. Ethics approval was obtained from the University of Stellenbosch and informed consent from respondents. A questionnaire was used to collect the data.

**Results:**

The hypothesis was accepted. The statistical association between affordability (*p* = < 0.01), accessibility (*p* = < 0.01) and the use of health services was found to be significant using the Chi-square (χ^2^) test.

**Conclusion:**

The study has shown how affordability and accessibility may influence the use of healthcare services. Accessibility is not only the distance an individual must travel to reach the health service point but more so the utilisation of these services. Continuous Quality Management should be a priority in healthcare services, which should be user-friendly.

## Introduction

Appropriate healthcare is a human right recognised universally in both national and international law (Pieterse 2006:473). The *Constitution of the Republic of South Africa*, *Act* 108 of 1996 (Republic of South Africa 1996), endorses the basic fundamental rights of individuals and emphasises the right to life and the right to health. The right to treatment includes the right to accessibility and affordability of medication which is described as an international law requirement (Yamin 2003). The Primary Health Care (PHC) Package for South Africa includes accessibility and affordability of health services to all South Africans (Department of Health 2000).

### Problem statement

The health status, accessibility, affordability and utilisation of healthcare seem to be related to both social and economic factors. It was therefore essential that a scientific investigation be undertaken to determine whether there was a relationship between accessibility, affordability and the use of health services by urban 22- to 50-year-old mixed-race (coloured) people of the Western Cape. This group, being the largest population group, contributes significantly to the economy of the Western Cape.

#### Objectives

The main aim of the study was to explore and describe the accessibility, affordability and the use of health services by the coloured population in the Western Cape.

#### Definition of key concepts

**Accessibility and affordability of health services:** The PHC Package for South Africa includes accessibility and affordability of health services to all South Africans (Department of Health 2000).

**Batho Pele principles:** Sesotho for ‘People First’. An initiative which aims to enhance the quality and accessibility of government services by improving efficiency and accountability to the recipients of public goods and services (Department of Public Service and Administration 1997).

**Economically active**, as defined by Statistics South Africa (2004), is a person of working age (15–65 years) who is available for work and is employed or unemployed.

#### Contribution to the field

The scientific evidence obtained in this study may enable policy makers in healthcare to take informed decisions in providing an accessible and affordable health service to the communities in an urban context in the Western Cape. This cross-sectional study spanned six densely-populated residential areas in the Northern and Eastern regions of the Cape Metropolitan area of the Western Cape, South Africa.

### Literature review

In his address on health reforms in the United States of America, President Obama indicated that health reform is a ‘moral obligation’ and, as described further by De Pinto (2009) in a poll of the American people, the Liberals (72%) believe that providing healthcare to all Americans is the government's ‘moral responsibility’. In the early 19th century, an English philosopher, Henry Spencer, issued a stern injunction that preservation of health was not a moral issue but a consequence of economics (*Lancet* Editorial 1996:1197). Economic growth has been associated with widening poor–rich disparities in health indicators (Houweling *et al*. 2005:1257). World-wide, less and less emphasis is placed on health as a priority. Houweling *et al*. (2005) emphasise that this effect may be remedied if public spending on health is increased. In 2004, the total gross domestic product (GDP) for health in Sudan was 4.2%; this declined to 3.6% in 2008, whilst the United States increased from 15.3% in 2004 to 15.7% in 2008. South Africa, being both a developing and a developed nation, has shown a steady decline in the relative expenditure on health from 9.2% in 2004 to 8.6% in 2008 (Trading Economics 2010).

The need to improve the average health status of populations was recognised by the member states of the World Health Organization (WHO) when the Alma Ata Declaration was adopted in the European Region (World Health Organization/United Nations International Children's Emergency Fund [UNICEF] 1978). A strategy of ‘Health for All’ was adopted. In addition, it was decided that by the year 2000, the differences in health status between countries and between groups within countries should be reduced by at least 25%. This could be achieved by improving the health of the disadvantaged nations and groups within countries. To address the ‘Gross inequality in the health status of the people, particularly between developed and developing countries, the WHO focused on PHC as the key to attain the goals of this strategy “Health for all by the year 2000”’ (WHO/UNICEF 1978). However, the United Nations (2000:1) at their Millennium Summit decided on more measurable goals with specific outcomes to ensure more healthy nations by 2015. With reference to these measurable goals with specific outcomes, Dr Margaret Chan, Director-General of the WHO, stated:

I do not believe we will be able to reach the Millennium Development Goals unless we return to the values, principles, and approaches of PHC … Decades of experience tell us that primary health care is the best route to universal access, the best way to ensure sustainable improvements in health outcomes, and the best guarantee that access to care will be fair. (International Council of Nurses 2008:2)

Despite its obvious advantages, the implementation of PHC has to overcome certain structural anomalies in countries like South Africa. Inequalities in healthcare between population groups of South Africa existed during the apartheid era and continue to exist both between and within many population groups, despite government efforts.

According to Kon and Lackan (2008) in their study on ethnic disparities in access to care in Post-Apartheid South Africa, they identified that black South Africans had the least access to medical care during the previous year (40.8%), followed by coloured people (22.9%), white people (10.9%) and Asians (6.9%). In a study conducted by Farmer and Ferraro (2005), individuals who are of a low socioeconomic standing are more likely to suffer from an increase in disease morbidity, higher mortality rates, lower life expectancy, fewer health screenings, reduced access to healthcare and lower rates of health insurance. Furthermore, financial strain as shown in a study was more likely to increase mortality in Black-American older women (Szanton *et al*. 2008).

According to Swinnerton (2006), poverty has been identified as being the greatest threat to health. The poor, who use public facilities, have less access to healthcare than the more affluent, who are often covered by medical aid insurance.

Yamin (2003) indicates that health services should not only be accessible but should include physical accessibility, as substantiated by the United Nations (2000):

[*h*]ealth facilities, goods and services must be within safe physical reach for all sections of the population, especially vulnerable or marginalized groups, such as ethnic minorities and indigenous populations, women, children, adolescents, older persons, persons with disabilities and persons with HIV/AIDS.

The use of health services may alter, depending on where the individual lives. Patient factors may be more important than supply factors in explaining the differential use of health services (Van der Heyden *et al*. 2003).

Bradshaw and Steyn (2001:103) identified a statistical relationship between the health status of the poor and that of the wealthy. They identified that chronic diseases such as asthma, smoking and abuse of alcohol were more prevalent amongst the poor than the rich; poorer areas are still plagued by a high burden of disease. To further substantiate this finding, Stellenberg, Welmann and Groenewald (2008) found a statistical relationship between the health status and the socioeconomic level of the coloured people of the Western Cape.

Bradshaw and Steyn (2001:iv) found that access to and quality of healthcare, measured by the treatment status of hypertension and use of medication in those suffering from asthma, was found to be worse amongst the poor. This is substantiated by the higher relative mortality burden of asthma and stroke amongst this group.

## Research methods and design

### Research design

The cross-sectional study used a descriptive, non-experimental research design with a quantitative approach to investigate and describe the relationship between the accessibility, affordability and use of health services by coloured people of both sexes in the Western Cape between the ages of 22 and 50 years. It was conducted amongst individuals in a well-defined population in order to assess both exposure and disease simultaneously. As described by Burns and Grove (2011:536), this approach ensures that all the characteristics of a single sample are described, including any phenomenon of interest and identification of the variables within said phenomenon.

### Hypothesis

The hypothesis was that there is a positive relationship between accessibility, affordability and the use of health services by the coloured people of an urban area in the Western Cape.

### Materials

A purposive convenient sample of 353 participants was drawn from the residential areas defined for the purpose of the study (Table 1). Guided by the statistician, the sample of 353 participants (0.6%) was drawn from a population of 63 004 economically-active people who lived in these suburbs (Statistics South Africa 1996).

**TABLE 1:** Residential areas (*N* = 353).

Area	Sample		Population	
	*n*	%	*n*	%
Bellville South	53	15.0	5303	8.4
Belhar	50	14.2	14119	22.4
Elsies River	80	22.7	20025	31.7
Kraaifontein	63	17.8	14427	23.0
Kuils River	51	14.4	945	1.5
Ravensmead	56	15.9	8185	13
Total	353	100.0	63004	100.0

### Context of the study

The study was conducted in six residential areas of the Northern and Eastern regions of the Cape Metropolitan area in the Western Cape, South Africa, which included all individuals of all socioeconomic levels.

The residential areas selected were categorised with the help of town planners of the municipalities concerned into upper–middle socioeconomic level, lower socioeconomic level (formal housing) and lower socioeconomic level (informal housing), as shown in Table 2.

**TABLE 2:** House structure classification (*N* = 353).

House structure	*n*	%
Upper–Middle economic	123	34.8
Formal: Lower economic	133	37.7
Informal: Lower economic	97	27.5
Total	353	100.0

The design of the sample maximised the chance that all social classes were fairly and equally represented, as the intention was to determine the association between the factors and not the probable size of the different factors in the population.

### Selection criteria

Respondents, who identified themselves as coloured persons as classified according to the population register during the apartheid era (1948–1994), who were 22 to 50 years old, economically active and gave written informed consent to participate, were included in the research study. The economically-active target group was chosen, as this target group by definition also included the unemployed individuals (Statistics South Africa 2004), who have the potential to contribute to the economy of the province.

The researcher advertised the study through the community leaders of the various residential areas. The purpose of the study was explained and they assisted in identifying individuals who met the criteria for participation in the study. A minimum of 50 persons who met the criteria in a particular residential area, as identified and classified for the study, were thus included in the study through convenience sampling.

### Pilot study

A pilot study was conducted to test the feasibility of the study; this included the methodology and administration procedure and the questionnaire so as to check for ambiguity and inaccuracies. A sample of 30 individuals (10%) of the initial planned sample of 300 of the main study was included in the pilot study. These participants were not included in the actual study.

### Data collection and instrumentation

The researcher collected the data personally with the help of two trained field workers (registered nurses) in a structured interview using a structured questionnaire. The questionnaire included questions about residential area, income, employment, types of health services used, accessibility and affordability of health services.

### Data analysis and interpretation of qualitative and quantitative data

A Statistical Analysis System (SAS) computer program was used to analyse the quantitative data. Descriptive and inferential statistics were applied in the analysis of the quantitative data.

The Chi-square (χ^2^) statistical test on a 95% confidence interval was applied to determine the significance level of the relationship between the accessibility, affordability and use of health services by the coloured people of the Western Cape in an urban area. This test was most appropriate for determining whether the two variables being examined are independent or related as described by Burns and Grove (2009:499–500).

The open questions were categorised, with common themes being identified and then quantified.

## Results

In this section, responses to questions related to the accessibility and affordability of health services are discussed.

### Type of health services used by respondents

The respondents used a variety of health services (Table 3). If their financial circumstances permitted, a private doctor was consulted, otherwise the comprehensive healthcare centre (CHC) or state health clinic was used.

**TABLE 3:** Type of health service respondents used (*N* = 353).

Health service	*n*	%
**State health clinic**		
Yes	110	31.2
No	243	68.8
**Comprehensive health service ‘day hospital’**		
Yes	200	56.7
No	153	43.3
**State hospital**		
Yes	165	46.7
No	188	53.3
**Private doctor**		
Yes	198	56.1
No	155	43.9
**Private hospital**		
Yes	79	22.4
No	274	77.6

The majority of the respondents used the state health facilities. The most commonly-used facilities were CHCs (*n* = 200; 56.7%), followed by private doctor (*n* = 198; 56.1%) and state hospitals (*n* = 165; 46.7%). CHCs were used by respondents of all socioeconomic levels: further analysis indicated that CHCs were used by 30 (24.4%) respondents from the upper–middle socioeconomic level, 90 (67.7%) from the lower socioeconomic level (formal housing) 80 (82.5%) from the lower socioeconomic level (informal housing). Only 79 (22.4%) of the respondents indicated that they used private hospitals. Private medical care is expensive and many of the respondents could not afford to use these services. Only 73 (20.7%) of the respondents had medical aid funds that allowed them to use a health service of their choice.

### Accessibility to health service

Table 4 shows that just over half (*n* = 203; 57.5%) of the respondents found the health services (including both public and private services) to be accessible. Using the Chi-square test (χ^2^), the statistical association between accessibility to health services and the use of health services was found to be significant (*p* = < 0.01)*.* The association between socioeconomic level and accessibility of health services was also found to be significant (*p* = < 0.01). Additional analysis indicated that 26 (32.5%) of the respondents from the upper-middle socioeconomic level, 66 (49.6%) from the lower socioeconomic level (formal housing) and 44 (45.4%) from the lower socioeconomic level (informal housing) found their health services to be inaccessible. Amongst those respondents who were employed, 78 (51.5%) found their health services to be inaccessible and 97 (48.5%) of the unemployed individuals responded likewise.

**TABLE 4a:** Health services accessibility (*N* = 353).

Accessibility to health services	*n*	%
Yes	203	57.5
No	150	42.5
Total	353	100

### Reasons for not finding health services to be accessible

As shown in Table 4, the 150 respondents for whom this question was relevant provided a variety of reasons for why they did not find the health services to be accessible. It is disconcerting to note that a number of the respondents (*n* = 44; 29.3%) indicated that staff displayed a negative attitude toward the patients and/or clients.

**TABLE 4b:** Reasons for finding health services inaccessible (*n* = 150).

Reasons for finding health services inaccessible (*n* = 150)	*n*	%
Staff (i.e., nurses, doctors and pharmacists) attitude negative toward patients/clients.	44	29.3
Poor service, including nursing, medical, pharmacy and emergency care.	17	11.3
No service exists for those who are prepared to pay a small fee for services.	7	4.7
Availability of services – no day hospital in one area, appointments essential even to attend a day hospital.	44	29.3
Overcrowding because of staff shortage.	26	17.3
Waiting period average 8–10 hours.	79	52.7
Unhygienic conditions in health institutions include all levels of service for example dirty toilets with faeces smeared on the walls and urine on floors.	17	11.3

Respondents felt that they were made to feel that a favour was being done for them, because the services were free. More than half (*n* = 79; 52.7%) of the respondents complained about the length of the waiting period, which averaged 10 hours. Respondents were required to be at the CHC by 06:00. If they arrived by 07:00, it might be too late to be attended to on that specific day.

When too many patients attended the health service on a particular day, rapid screening was done by the registered professional nurse and even if a respondent was there at 06:00, he or she could be told to come back the next day. This could have financial implications such as losing a day's wages. The patients might also not have money for transport to return the next day. In addition, frequent days off work could result in conflict between employer and employee and consequent loss of employment. An ultimate result of these factors might be delayed health-seeking behaviour.

Some of the respondents (*n* = 17; 11.3%) also reported unhygienic conditions (such as unhygienic toilets) in state health institutions, which made the long waiting hours unpleasant.

Table 4 shows that 44 (29.3%) of the respondents reported that the availability of services was so poor that an appointment system was practised at certain CHCs so as to alleviate overcrowding. The members of the public were turned away if they did not have appointments. There were no CHC services in one of the residential areas, thereby forcing the residents to use a taxi and a train to get to the recommended CHC. Inaccessibility as a result of geographical distance can also result in delayed health-seeking behaviour, which could aggravate health problems; an example of this is a respondent who developed a foot infection after a motor vehicle accident, but who could not get to a CHC because of unemployment and eventually, nine months later, required urgent medical attention and hospitalisation.

Many similar cases of delays in seeking medical assistance may occur as a result of health services being inaccessible. Delayed health-seeking behaviour takes its toll, not only on the health status of the individual, but also in terms of becoming a financial burden for the state.

### Affordability of health services

The majority of the respondents (*n* = 184; 52.1%) found their health services to be affordable; however, most of this group were not paying for their health services. An analysis according to socioeconomic level indicates that the respondents who found the health services to be most affordable were from the lower socioeconomic (informal) level (*n* = 63; 60.8%), followed by the upper-middle socioeconomic level (*n* = 72; 58.5%) and the lower socioeconomic (formal) level (*n* = 53; 39.9%). All of the respondents who found that health services were not affordable indicated that they were forced to use the CHC because private doctors were too expensive. The statistical association between affordability and the use of health services was significant (*p* = < 0.01).

### Respondents who have a medical aid fund

Only 73 (20.7%) of the respondents belonged to a medical aid fund. This explains the overcrowding at CHCs, as 200 (56%) of the respondents were using the services of a CHC, as is shown in Table 3. A breakdown of the figures shows that respondents who had a medical aid fund came from all socioeconomic levels: 58 (47.2%) from the upper–middle socioeconomic level, 9 (6.8%) from the lower socioeconomic (formal) level and 6 (6.2%) from the lower socioeconomic (informal) level.

The association between the affordability of medical services and belonging to a medical aid fund was of statistical significance (*p* = < 0.01), as tested with the Chi-square test. Further analysis also showed a statistically-significant association between the residential area and the respondents who had a medical aid (*p* = < 0.01).

### Respondents’ reasons for not using their health services

In response to an open-ended question, the respondents gave the following reasons for not using their health services: 28 (51.9%) indicated that access problems, such as long queues of people and long waiting hours at CHCs, prevented them from using their health services; 15 (27.8%) had no taxi and/or train fare to get to the CHC; and 11 (20.3%) indicated that they did not require such a service.

The introduction of a payment service to improve the health service was suggested by 17 (15%) of the respondents, as the country is already burdened with existing financial constraints and cannot afford to give a free health service to the majority of its citizens. Re-evaluation of this policy should thus occur. Payment for services might not only benefit the country, but the patients as well, as they would learn to take responsibility for their own health. Cognisance should be taken of the valuable input made by patients, as they are the consumers of such a service.

The hypothesis set for this research was accepted as the research showed that there was a positive relationship between accessibility, affordability and the use of health services by the coloured people in an urban area of the Western Cape.

## Ethical considerations

Ethical approval to conduct this research study was obtained from the Ethics Committee of the Faculty of Medicine and Health Sciences at Stellenbosch University. Confidentiality and anonymity were assured and informed written consent was obtained from all participants. Access to the data was limited to the researcher and data were kept in a locked room for a period of five years.

## Trustworthiness

### Validity and reliability

A pilot study was conducted to test the feasibility of the study; this included pre-testing the instrument for inaccuracies and ambiguity in support of its validity and reliability. Experts in research methodology, statistics and computer science evaluated the instrument. In addition, the content of the instrument was evaluated by a nursing science specialist, a sociologist and members of the ethics and postgraduate committee. Reliability was further assured by using two well-trained assistants together with the researcher and using standard instructions for all participants for the data collection.

## Discussion

This study showed that inaccessibility to health services affects members of all socioeconomic levels. Some of the reasons for inaccessibility identified in the interviews were that the conditions in state institutions were appalling and unhygienic, the quality of service was poor and services were far from their places of residence.

One suburb had no CHC and the respondents of this area had to take a taxi and/or train to get to the nearest health service. Improving access, equity, efficiency and sustainability is part of the Department of Health's mission (Department of Health 2004). The 2010 Service Plan of the Western Cape Government indicates that the distance between health clinics in the densely-populated Cape Metropolitan District will be a walking distance of three to four kilometres (Provincial Government of the Western Cape 2006).

The fact that 42.5% of the respondents did not find the services accessible is problematic, as inaccessibility of health services may lead to delayed health-seeking behaviour, which could increase the morbidity and mortality in this sector of the population.

The PHC policy indicates that the population should use the first level of healthcare which in the Cape Metropolitan area is the ‘day hospital’ or CHC. If clients bypass this, they will be penalised by having to pay at the next level of service.

According to Braveman and Gruskin (2003), a commitment to health does not necessarily imply a commitment to reducing poverty, especially given the strong and pervasive links between poverty and health. The poor have less access to health services than the wealthy (Peters *et al*. 2008; Stellenberg *et al*. 2008). Nevertheless, it is suggested that:

[*s*]trategies that narrowly focus on poverty and health without the broader perspectives offered by equity and human rights may fail because they do not take into consideration the key factors that often influence the relationship between poverty and ill-health. (Braveman & Gruskin 2003:540)

## Limitations

The limitation identified in this study was that it only covered the coloured people of the Western Cape in an urban area. Further investigation should be undertaken between all population groups in all areas.

## Recommendations

Despite the disparities of the past, bringing about equity in healthcare must be based on scientific evidence in order to provide appropriate healthcare and ensure cost-effectiveness. The recommendations in this section are made as a result of the findings of this study.

### Review of the free healthcare policy

A review of the free medical service is recommended. A small payment will promote a sense of responsibility and free medical service should then be reserved for senior citizens, the severely disabled and children.

### Improvement of the health services at clinics

CHCs are currently required to serve the majority of the communities. The study showed that 56.7% of the respondents used CHCs, but the number of patients reporting to CHCs will in all likelihood increase rather than decrease as a result of the global economic crisis.

A significant portion of respondents (66.4%) indicated that the services were poor, aggravated by long waiting times and unhygienic conditions. Many respondents (29.3%) complained about the attitude of nurses, who made patients feel that they were doing them a favour because of the free medical care. In addition, 17.3% of respondents indicated that the CHCs are overcrowded because there is a staff shortage. These problems could explain the complaints of patients regarding the negative attitude of the staff.

Functioning in the trying circumstances described above is not conducive to quality healthcare delivery as the limited staff are pressurised to get through large numbers of patients.

### Recommendations with respect to the health services

#### Personnel

The nursing, medical and pharmaceutical staff complements should receive urgent attention and be increased as required in order to meet the needs of the population. All hospital staff should be involved in strategic planning.

#### Organisational aspects

The 24-hour service already existing at some CHCs should be extended to all CHCs. Alternatively, the hours that those CHCs that do not offer 24-hour service should be extended so as to enable patients to be attended to after hours. Providing an adequate service to the people will allow the patient to be at work during the day and not lose a day's salary or wage. The number of patients that crowd a CHC from as early as 06:00 could also be prevented.

The introduction of specific clinics, such as hypertension or diabetic clinics, should be scheduled for mornings or afternoons on certain days. These sessions may also be used to address groups of patients on specific health talks or to show videos of common health problems related to the disease in question.

Services for patients who do not have appointments should be introduced. It is unethical to turn patients away.

In addition, services should be available in all suburbs. According to the PHC policy, healthcare services should be available in all communities (Department of Health 2000).

Finally, mobile clinics should be introduced in informal settlements to meet the high demand for services.

#### Clinical environment

The hygiene of the institutions and especially the public toilets should be monitored. Staff should be appointed specifically for these tasks; for example, an hourly roster system should be introduced for the cleaning of toilets.

#### Quality assurance programmes

Introduction of quality assurance programmes, regular audits and accreditation of the state services is recommended. It is essential to monitor and evaluate specific indicators which may contribute to a positive or negative service. Continuous Quality Management should also be a priority in the state service.

#### Staff in-service training

Staff should receive continuous training in quality management in health service delivery, which should include the Patients Rights Charter (Department of Health 2004) and Batho Pele Principles (Department of Public Service and Administration 1997).

## Conclusion

In conclusion, every citizen has a right to have access to healthcare services (Republic of South Africa 1996). This discussion has shown how affordability and accessibility can influence the use of healthcare services. Accessibility is not only the distance an individual must travel to reach the health service point but more so the utilisation of these services. Services should be user-friendly to the consumer. The introduction of the eight Batho Pele-‘People First’ principles provide a policy framework and a practical implementation strategy for the transformation of the Public Service so as to ensure that the rights of patients to effective and efficient healthcare are met (Department of Public Service and Administration 1997). However, these principles should be evaluated at regular intervals by means of, amongst others, patient satisfaction surveys.
